# Supplementary material for: Development of a Protein Scaffold for Arginine Sensing Generated through the Dissection of the Arginine-Binding Protein from Thermotoga maritima
Source: Int J Mol Sci. 2020 Oct 12;21(20):7503. doi: 10.3390/ijms21207503 (PMC7589609; doi:10.3390/ijms21207503)
Supplement: Supplementary file 1 [file ijms-21-07503-s001.pdf]

Supplementary Materials

	1 … 50
TmArgBP	**MKKLLAVSIL LVSIVIFSG**A IDEIKSRGYL LVGLSADFPP FEFVDENGNI
$TmArgBP^{20-233\_F76W}$	A IDEIKSRGYL LVGLSADFPP FEFVDENGNI
$D1^{F57W}$	A IDEIKSRGYL LVGLSADFPP FEFVDENGNI
	51 … 100
TmArgBP	VGFDVDLAKE IARRLGVELK IVDMTFDGLI PSLLTKKIDV IISGMTITEE
$TmArgBP^{20-233\_F76W}$	VGFDVDLAKE IARRLGVELK IVDMT**W**DGLI PSLLTKKIDV IISGMTITEE
$D1^{F57W}$	VGFDVDLAKE IARRLGVELK IVDMT**W**DGLI PSLLTKKIDV IISGMTITEE
	101 … 150
TmArgBP	RKKVVAFSDP YFDAGQVIVV RKDSDFRPKT YEDLVGKTVA VQIGTTGDIE
$TmArgBP^{20-233\_F76W}$	RKKVVAFSDP YFDAGQVIVV RKDSDFRPKT YEDLVGKTVA VQIGTTGDIE
$D1^{F57W}$	RKKVVAFSDP YFDA**GGGGSG** --------------------------------
	151 … 200
TmArgBP	VSKYDGIKVV RFDKFTDAFL ELKRGRADAV VLDSATARAF VAKNPDLVIS
$TmArgBP^{20-233\_F76W}$	VSKYDGIKVV RFDKFTDAFL ELKRGRADAV VLDSATARAF VAKNPDLVIS
$D1^{F57W}$	--
	201 … 246
TmArgBP	SGVLSSEQYG IAVRKEDTDL LEFINSVLRE LKKSPYDVLI EKWFSE
$TmArgBP^{20-233\_F76W}$	SGVLSSEQYG IAVRKEDTDL LEFINSVLRE LKK
$D1^{F57W}$	------EQYG IAVRKEDTDL LEFINSVLRE LKK**LE HHHHHH**

Figure 1. Amino acid sequence of the wild-type TmArgBP and of the two protein variants $TmArgBP^{20\text{-}233\_F76W}$ and $D1^{F57W}$ characterized in this study. The transmembrane N-terminal fragment (residues 1-19) of TmArgBP is reported in bold. The mutation site F→W is highlighted in yellow. The D1 domain of TmArgBP is made of fragments that are non-consecutive in the sequence of the wild-type protein (residues 20–114 and 207–233) and that were artificially connected by a GGGGSG flexible linker indicated in red. Finally, the C-terminal His-tag present in $D1^{F57W}$ is reported in green.

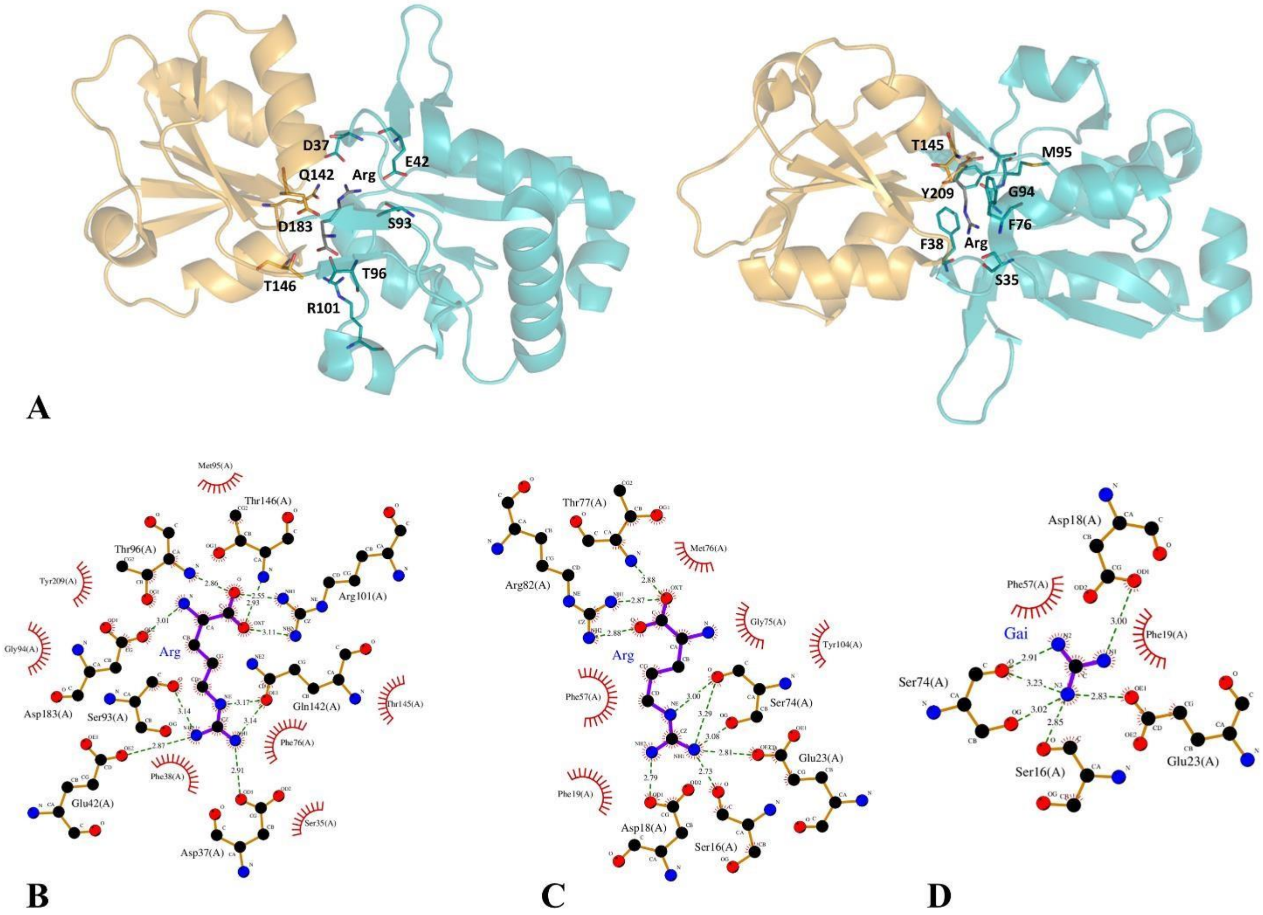

Figure 2. Cartoon representation of the crystal structure of the arginine-bound form of TmArgBP$^{20\text{-}233}$ (PDB ID: 6GGV). (**A**) The residues of the pocket that interact with the arginine through. H-bonding (left panel) or hydrophobic (right panel) interactions are represented as sticks. D1 and D2 domains are colored in cyan and orange, respectively. Network of the interactions established by Arg with the protein residues: (**B**) TmArgBP$^{20\text{-}233}$ (PDB ID: 6GGV), and (**C**) D1 (PDB ID: 6GPC). Interactions of the GuH^{+} ion with the D1 domain (PDB ID: 6Y16) (**D**). Panels B-D have been generated using the program LIGPLOT.

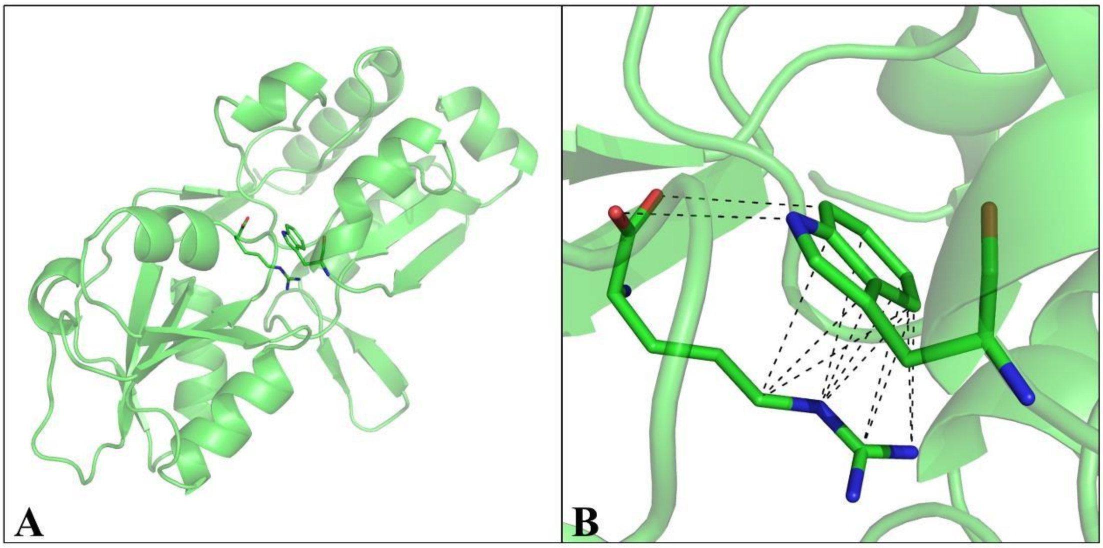

Figure 3. Modelling of the Phe76Trp mutation in the arginine-bound form of TmArgBP$^{20\text{-}233}$ (**A**). Evidence for limited but significant steric clashes: distances lower than 4.0 Å are shown (**B**).

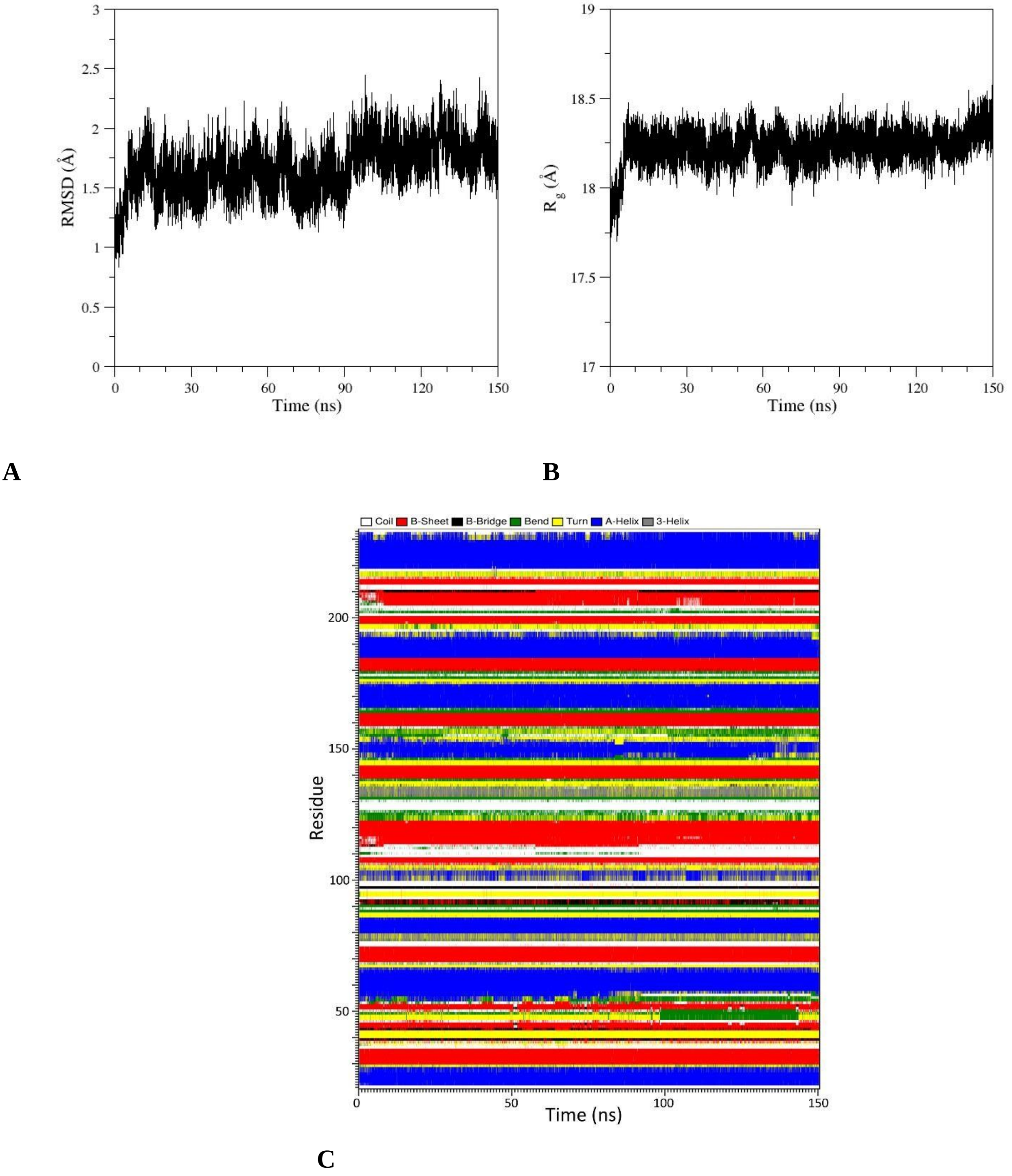

Figure 4. Stability of TmArgBP$^{20\text{-}233_F76W}$ in the MD simulation. Time evolution of RMSD values of trajectory structures compared to the starting crystallographic model (**A**), radius of gyration R_g (**B**), and secondary structure content (**C**) in the MD simulation of TmArgBP$^{20\text{-}233_F76W}$. The protein C^α atoms were considered in the RMSD and R_g calculations.

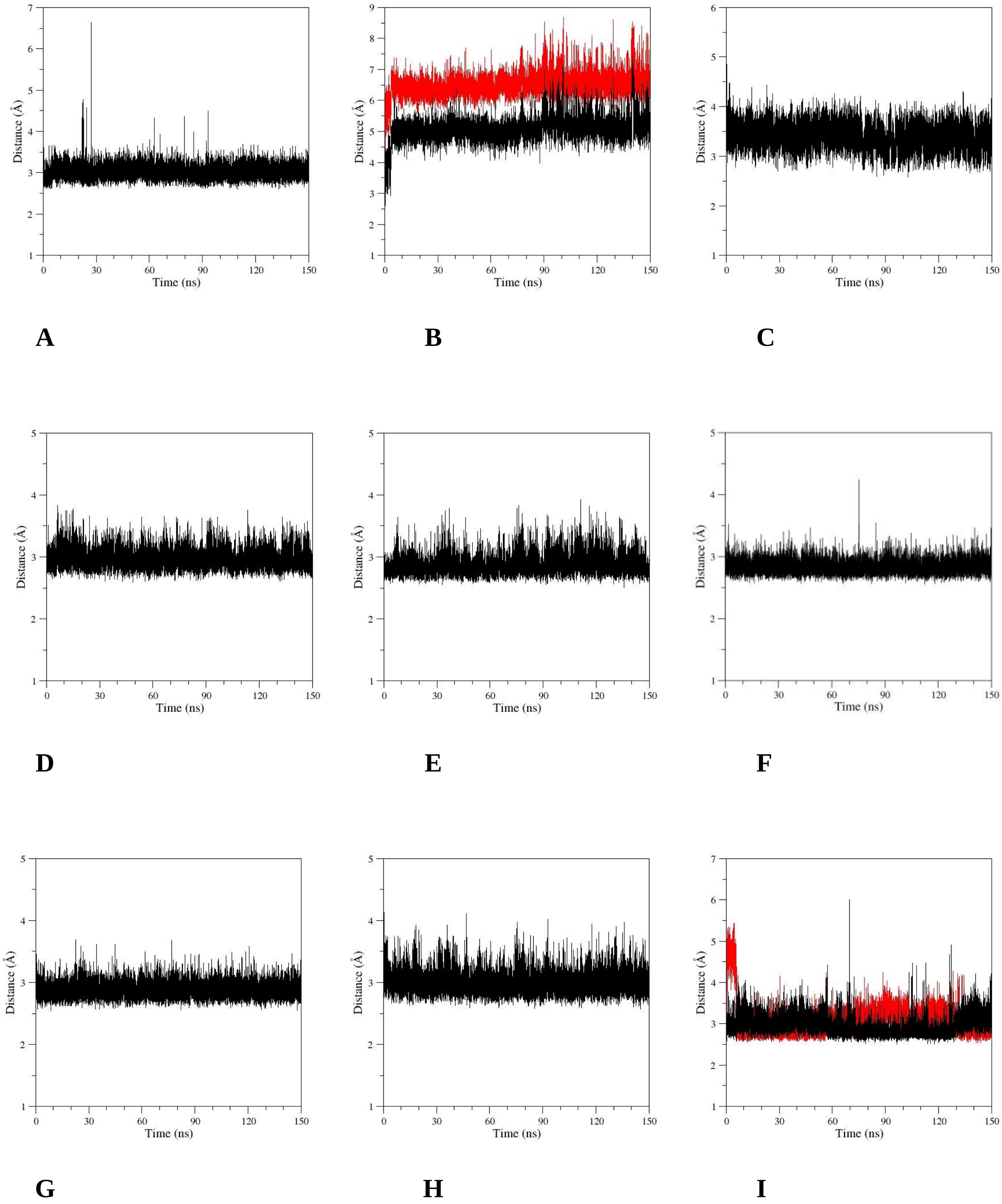

Figure 5. Time evolution of the distances between pairs of atoms involved in the formation of Hbonding interactions in the MD simulation of TmArgBP$^{20\text{-}233_F76W}$: Asp37$^{O\delta1}$-Arg$^{N\eta1}$(**A**), Glu42$^{O\varepsilon2}$(black)/Glu42$^{O\varepsilon1}$(red)-Arg$^{N\eta2}$(**B**), Ser93^{O}-Arg$^{N\eta2}$(**C**), Thr96^{N}-Arg^{O} (**D**), Arg101$^{N\eta1}$- Arg^{O}(**E**), Arg101$^{N\eta2}$- Arg^{OXT}(**F**), Gln142$^{O\varepsilon1}$-Arg$^{N\eta1}$ (**G**), Thr146^{N}-Arg^{OXT} (**H**),.Asp183$^{O\delta2}$(black)/Asp183$^{O\delta1}$(red)-Arg^{N} (**I**).

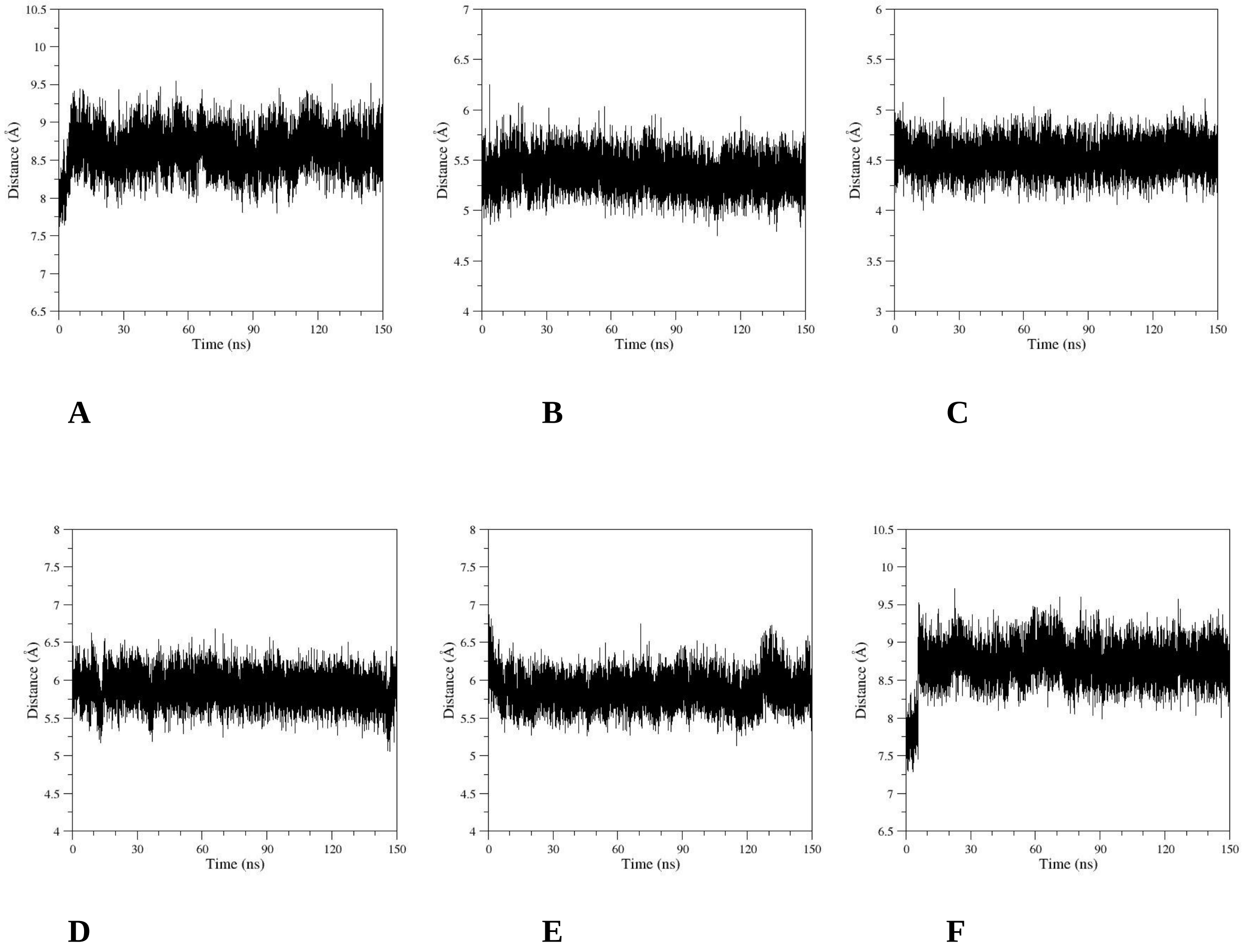

Figure 6. Time evolution of the distances between the centers of mass of the arginine ligand and the residues involved in the hydrophobic interactions in the MD simulation of TmArgBP$^{20\text{-}233_F76W}$: Ser35 (**A**), Phe38 (**B**), Gly94 (**C**), Met95 (**D**), Thr145 (**E**), and Tyr209 (**F**).

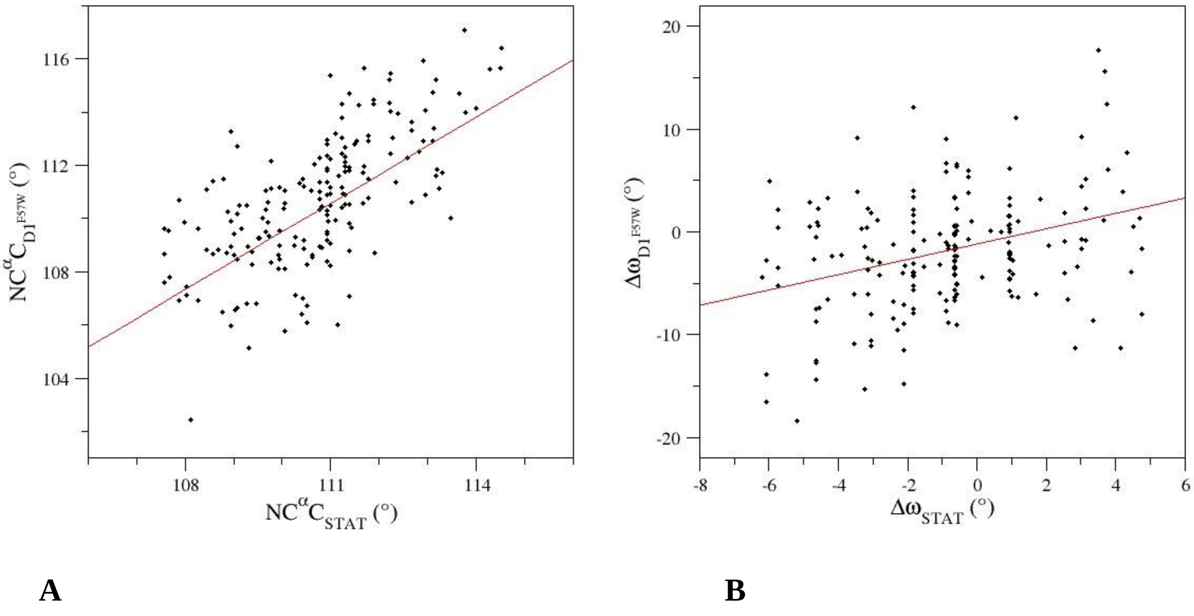

Figure 7. Validation of the variability of geometrical parameters of the protein backbone in the crystallographic structure of TmArgBP D1^{F57W}: the NC$^{\alpha}$C bond angle (**A**) and the deviation from the peptide bond planarity $\Delta\omega=(\omega\text{-}180°)\text{mod}360°$ (**B**). The regression analysis has been carried out by plotting the NC$^{\alpha}$C or $\Delta\omega$ values of residues of the final model against the average NC$^{\alpha}$C or $\Delta\omega$ values of residues adopting the same (φ, ψ) conformation obtained from datasets of X-ray PDB structures solved at high resolution (release of March 2016). These protein structures were selected with the PISCES culling server (http://dunbrack.fccc.edu/PISCES.php) applying the following criteria: resolution better than 1.6 Å for bond angles and 1.2 Å for dihedral angles, R-factor ≤ 0.20, and sequence identity ≤ 25%. Further selections were carried out at residue level by excluding residues for which the ratio between the average backbone B-factor (atomic displacement parameter) of the residue and the same parameter computed considering the entire chain was higher than 1.3. These databases contain 3,291 (bond angles) and 799 (dihedral angles) non-redundant protein chains. The regression lines are shown. The parameters (correlation coefficient and p-value) of the linear fitting are reported in **Table S1**.

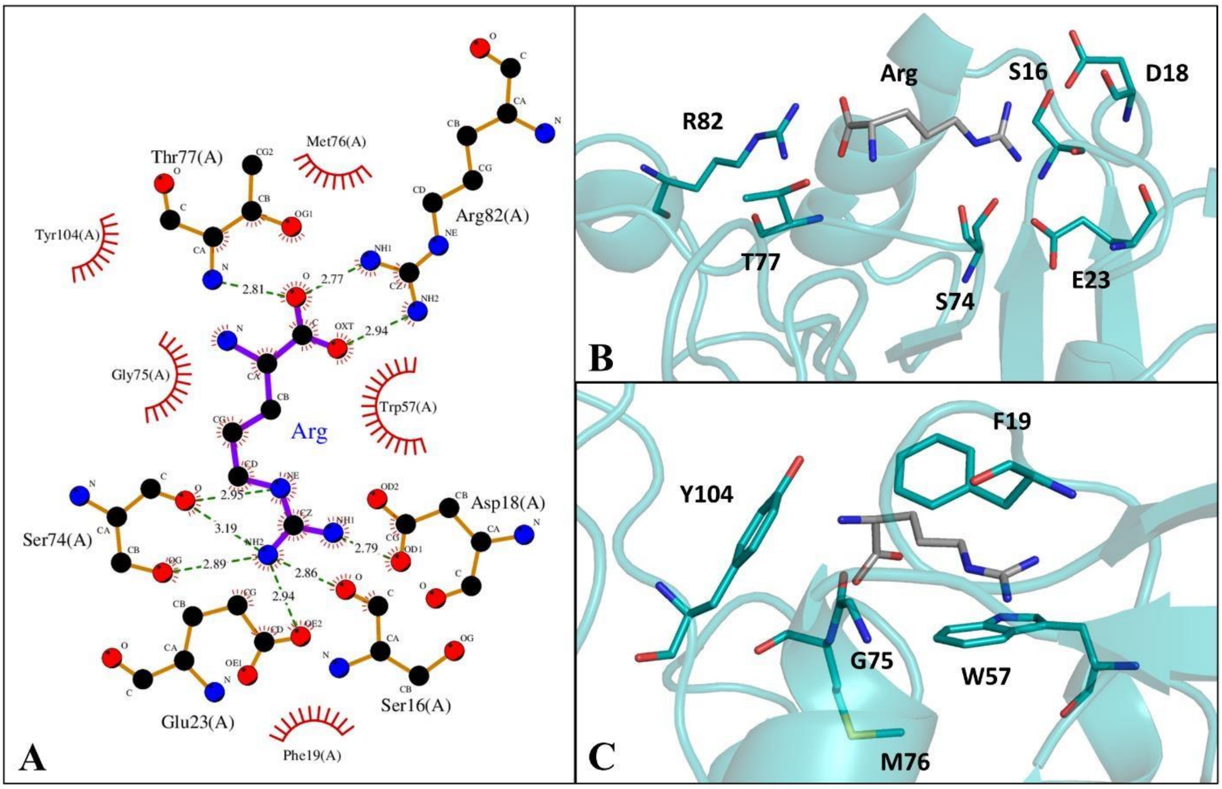

Figure 8. Network of the interactions established by Arg with the protein residues in the crystal structure of D1^{F57W} from TmArgBP obtained with the LIGPLOT program (**A**). The residues of the pocket that interact with the arginine through H-bonding (**B**) or hydrophobic (**C**) interactions are represented as sticks.

Table 1. Results of the regression analysis of the variability of several bond angles, the deviation from peptide bond planarity $\Delta\omega$, and the carbon carbonyl pyramidalization θ_C. The geometrical parameters computed for the structure of TmArgBP D1^{F57W} were compared to those derived from well-refined protein structures (see text and legend of Figure S7).

Geometrical Parameter	Correlation Coefficient	p-Value
$NC^{\alpha}C$	0.66	$<10^{-6}$
$NC^{\alpha}C^{\beta}$	0.12	0.12
$C^{\beta}C^{\alpha}C$	0.31	$2.3*10^{-5}$
$C^{\alpha}CO$	0.30	$2.7*10^{-5}$
CαCN+1	0.36	$<10^{-6}$
OCN_{+1}	0.19	0.011
C-1NCα	0.45	$<10^{-6}$
$\Delta\omega$	0.34	$3*10^{-6}$
θC	0.25	$6.6*10^{-4}$

Table 2. Structural comparison of TmArgBP D1^{F57W} with the structure of the ligand-free (lf) and arginine-bound (ab) forms of the wild-type TmArgBP, the truncated form TmArgBP$^{20-233}$, and D1 in terms of C$^{\alpha}$-based RMSD values. Chain A of TmArgBP D1^{F57W} has been considered in the calculation.

Protein	PDB Entry	RMSD (Å)
lf-TmArgBP chain A	4PRS	0.471
lf-TmArgBP chain B		0.506
ab-TmArgBP chain A	4PSH	0.433
ab-TmArgBP chain B		0.409
lf-TmArgBP20-233	6GGP	0.455
ab-TmArgBP$^{20-233}$ chain A	6GGV	0.500
ab-TmArgBP$^{20-233}$ chain B		0.548
lf-TmArgBP D1	6GPD	0.315
ab-TmArgBP D1 chain A	6GPC	0.310
ab-TmArgBP D1 chain B		0.312

Table 3. Atoms of the arginine bound to TmArgBP D1^{F57W} that are closer than 4.0 Å to atoms of W57 side chain.

Interacting atoms		Distance (Å)
Arginine	W57	
NE	CZ3	3.27
CZ	CE3	3.54
NE	CE3	3.56
CD	CZ3	3.61
CZ	CZ3	3.66
NH1	CE3	3.67
O	CH2	3.74
CG	CH2	3.75
C	CZ2	3.77
O	CZ2	3.78
CD	CE3	3.83
CD	CH2	3.89
CG	CZ3	3.94
C	CH2	3.97
NE	CH2	3.99
